# AI-based MRI auto-segmentation of brain tumor in rodents, a multicenter study

**DOI:** 10.1186/s40478-023-01509-w

**Published:** 2023-01-14

**Authors:** Shuncong Wang, Xin Pang, Frederik de Keyzer, Yuanbo Feng, Johan V. Swinnen, Jie Yu, Yicheng Ni

**Affiliations:** 1 Biomedical Group, Campus Gasthuisberg, KU Leuven, 3000 Leuven, Belgium; 2 Department of Radiology, University Hospitals Leuven, KU Leuven, Herestraat 49, 3000 Leuven, Belgium; 3 Faculty of Economics and Business, KU Leuven, 3000 Leuven, Belgium

**Keywords:** Artificial intelligence, Brain malignancy, MRI, Segmentation, Rodent

## Abstract

Automatic segmentation of rodent brain tumor on magnetic resonance imaging (MRI) may facilitate biomedical research. The current study aims to prove the feasibility for automatic segmentation by artificial intelligence (AI), and practicability of AI-assisted segmentation. MRI images, including T2WI, T1WI and CE-T1WI, of brain tumor from 57 WAG/Rij rats in KU Leuven and 46 mice from the cancer imaging archive (TCIA) were collected. A 3D U-Net architecture was adopted for segmentation of tumor bearing brain and brain tumor. After training, these models were tested with both datasets after Gaussian noise addition. Reduction of inter-observer disparity by AI-assisted segmentation was also evaluated. The AI model segmented tumor-bearing brain well for both Leuven and TCIA datasets, with Dice similarity coefficients (DSCs) of 0.87 and 0.85 respectively. After noise addition, the performance remained unchanged when the signal–noise ratio (SNR) was higher than two or eight, respectively. For the segmentation of tumor lesions, AI-based model yielded DSCs of 0.70 and 0.61 for Leuven and TCIA datasets respectively. Similarly, the performance is uncompromised when the SNR was over two and eight respectively. AI-assisted segmentation could significantly reduce the inter-observer disparities and segmentation time in both rats and mice. Both AI models for segmenting brain or tumor lesions could improve inter-observer agreement and therefore contributed to the standardization of the following biomedical studies.

**Supplementary Information:**

The online version contains supplementary material available at 10.1186/s40478-023-01509-w.

## Introduction

Malignant brain tumor, both primary and metastatic lesions, have long been an unsolved clinical problem, due to highly progressive characteristics and limited therapeutics [19, 20, 23, 24]. Development of novel therapeutics for brain tumor is an urgent need. Due to its capabilities for precise intracranial localization and superb soft tissue contrast, MRI represents a powerful tool to provide in vivo and non-invasive visualization of brain tumor anatomy and functionality in both clinics and animal research [23]. Quantitative imaging analyses including radiomics, MRI-based surgical planning, and radiotherapy design are highly dependent on the proper segmentation of the brain tumor lesions, which is conventionally finished by well-trained radiologists [17]. However, segmentation by humans is time-consuming and, furthermore, inter-observer variability may be introduced during this process.

Empowered by state-of-the-art artificial intelligence (AI) algorithm, automatic semantic segmentation of region of interest (ROI) becomes possible with reduced inter-observer disparity [2]. U-Net is dedicated convolutional neural network (CNN) for biomedical image segmentation by an encoder/decoder structure that integrates multiscale information and shows better gradient propagation [18, 25]. The 2D U-net can capture MRI image features and return a probability map for each pixel to be classified as ROI or not. However, it only captures in-plane image texture, leaving behind trans-plane information [9, 18]. To overcome this, a “2.5D” model was adopted by including few neighboring slices as a compromised manner and proven to show improved performance as expected [5]. Whereas, 3D U-net takes into account of the cross-plane information natively, aiming to simulate the way how radiologists interpret medical images [3].

In preclinical imaging studies, rodent brain extraction is often achieved by manually drawing brain masks for each slice. Previously, Hsu et al. proposed AI models for segmentation of brain in T2-weighted turbo-spin-echo structural MRI and T2*-weighted echo-planar-imaging functional MRI images from normal mice model based on 2D U-net and 3D U-net architectures [9, 10]. However, these models fail to reproduce properly in the oncological settings due to the presence of brain tumor and tumor-accompanying signs like ventriculomegaly and distorted brain morphology. Currently there is no study proposing models dedicated for the segmentation of brain tumor in rodents. To this end, this study is designed to explore: (1) feasibility of AI-based segmentation of tumor-bearing brains and brain tumors in rodents; and (2) practicability of AI-assisted segmentation. To make such tasks more applicable, we have developed two AI-based models, which finish the semantic segmentation in a stepwise way. The model 1 is responsible for segmentation of tumor bearing brain from head and neck region, whereas the model 2 is for segmentation of tumor(s) from the brain. Hopefully, the successful development of these models may reduce inter-observer disparity, save researchers’ time, and automate the processing of MRI data from brain volumetric dataset.

## Method

**Fig. 1:**
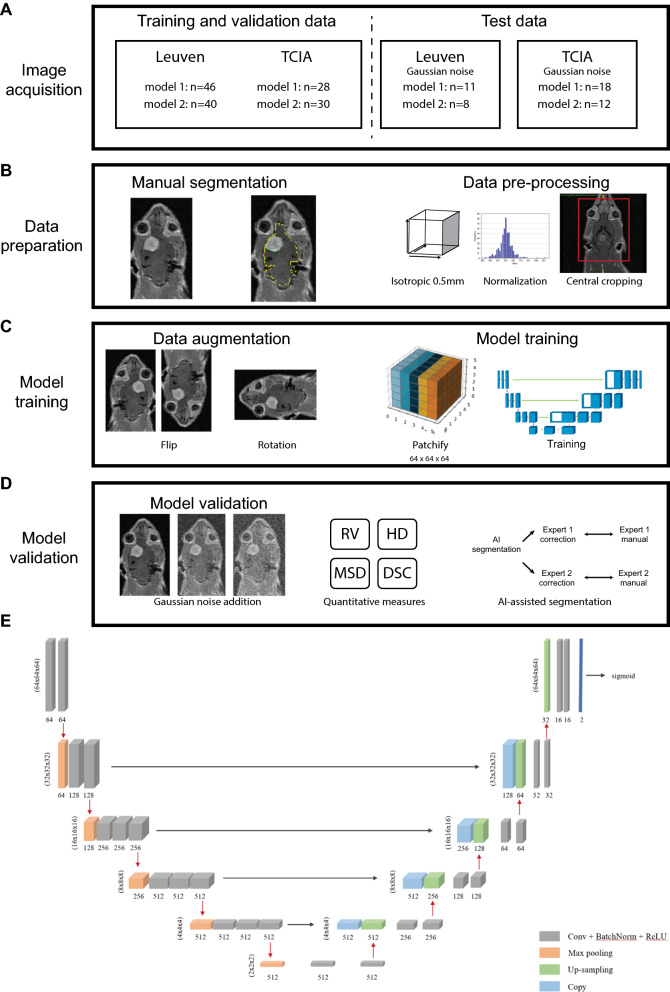
Flow chart and 3D U-Net architecture for current study. Collection and allocation of both data for model training, validation and test (**A**). All these data were manually segmented and pre-processed (**B**), followed by data augmentation and model training (**C**). The trained models were challenged by images with Gaussian noise added, measured quantitatively (**D**). AI-assisted segmentation was demonstrated based on ground truth by two radiologists (**D**). The same 3D U-Net architecture for training the two models was shown (**E**). Abbreviations: AI: artificial intelligence; RV: relative ratio; HD: Hausdorff distance; MSD: mean surface distance; DSC: Dice similarity coefficient

The study was executed as shown in Fig. 1, including image acquisition, data preparation, model training and model validation.

### Collections of datasets

For the Leuven dataset, the animal model of metastatic brain tumor was constructed with proper laboratory animal care, after ethical committee approval of KU Leuven (P046/2019) (Fig. 1A). Rat rhabdomyosarcoma cell line, kindly provided by Lab of Nanohealth and Optical Imaging group in KU Leuven, was cultured with 10% FBS and 1% penicillin/streptomycin at 37 °C in a 5% CO_2_ atmosphere in DMEM (Gibco, USA). The contamination of mycoplasma was excluded by e-Myco PCR kit (Boca Scientific, USA). The cell line was chosen based on the following considerations. Firstly, it is natively compatible with immune competent WAG/Rij rats, where reproduction of cancer-immunity interaction is possible. Secondly, its derived animal model exhibits similar MRI manifestation with clinical patients [22]. Thirdly, we aimed at developing models for brain or brain tumor segmentation, instead of elaborating on the biological disparity between different types of brain metastasis. The brain metastasis model was induced by surgical implantation, as published before [22].

MRI scans were performed using a 3.0T magnet (MAGNETOM Prisma; Siemens, Erlangen, Germany), with a 16-channel phase array wrist coil under gas anesthesia of a mixture of 3% isoflurane, 80% air and 17% oxygen, with the MRI sequences optimized from clinically used ones (Table 1). To ensure generalizability and translation potential, the commonly used sequences were adopted, including T1 weighted imaging (T1WI), T2 weighted imaging (T2WI) and contrast-enhanced T1 weighted imaging (CE-T1WI). These sequences provide high-resolution and not-distorted anatomical information in the brain. To increase the generalizability of the model, cases with various sizes of tumors, cases with/without ventriculomegaly, cases with intra-tumoral necrosis were included. Cases with missing/unsatisfactory MRI images were excluded.

**Table 1:** Summary of MRI scanning parameters

Protocol	Leuven-T2WI	Leuven-T1WI	TCIA-T2WI	TCIA-T1WI
Sequence	SE	SE	SE	GR
T_E_	147	700	100	4.188
T_R_	3000	9.0	6132	20.5
Voxel size (mm^^3^)	0.29*0.29*0.43	0.21*0.21*0.43	0.12 × 0.12 × 0.50	0.12 × 0.12 × 0.25
Matrix size	192 × 192 × 80	256 × 256 × 80	256 × 256 × 30	256 × 256 × 48
Field of view (mm)	55 × 55	55 × 55	30 × 30	30 × 30
Plane	Coronal	Coronal	Transverse	Transverse
Average	1	2	8	4
Flip angle	120	120	90	25
Band width	260	435	152	169
Magnetic field	3T	3T	3T	3T
Animal species	Rats	Rats	Mice	Mice

T2WI: T2 weighted imaging; T1WI: T1 weighted imaging; T_E_: echo time; T_R_: repetition time

The Cancer Imaging Archive (TCIA) dataset consists of MRI images (Philips 3.0T magnet, the Netherlands) from genetically engineered mouse models of high-grade astrocytoma, including glioblastoma multiforme and surgically implanted orthotopic model based on U87 cell lines. In the genetically engineered mouse models, the most dysregulated networks in glioblastoma multiforme including RB, KRAS and PI3K signaling are perturbed. These genetic aberrations induce development of mouse high-grade astrocytoma like that in humans. Thus, the TCIA dataset is more diverse in terms of tumor induction methods, pathological and genetic profiles. Two out of 48 cases were excluded from the TCIA dataset due to incompleteness of sequences (Fig. 1). Cases with ambiguous tumor lesion were excluded from model training.

A total of 46 cases from TCIA and 57 cases from Leuven dataset respectively were included. For model 1 that is responsible for segmentation of tumor bearing brain, a total of 57 cases were collected in KU Leuven, with 46 for training and 11 for validation. A total of 46 cases were collected in TCIA, with 28 for training and 18 for validation. For model 2 that is responsible for segmentation of brain lesions, a total of 48 cases were collected in KU Leuven, with 40 for training and 8 for validation. 42 cases were collected in TCIA, with 30 for training and 12 for validation.

### Manual segmentation

Generation of ground truth for both Leuven and TCIA datasets, facilitated by intensity-based thresholding and region-growing algorithms, was finished in ITK-SNAP (http://www.itksnap.org) by two co-authors, Yuanbo Feng, and Yicheng Ni, with more than 10 years of experience in experimental and clinical radiology (Fig. 1B) [26]. The segmentations of the brain and tumor were finished separately. Segmentation of brain was mainly based on T2WI and propagated to other sequences. Tumor segmentation was mainly based on CE-T1WI, with reference information from other sequences. For each segmentation task (either brain or tumor), Yicheng Ni and Yuanbo Feng performed segmentation independently, and consensus was achieved after discussion whenever there was a disagreement.

### AI model architecture

We adopted a stepwise solution for the segmentation tasks: firstly, developing a model for segmentation of tumor-bearing brain from the images of head and neck region; and secondly, developing a model for segmentation of tumor from the brain images for both datasets (Fig. 1C). These models are named as model 1 (segmentation of tumor-bearing brain) and model 2 (segmentation of brain tumor). The adoption of step-wise solution is based on consideration of future applications. Segmentation of only the brain tissue, namely skull stripping, highlights brain morphology. In quantitative imaging analyses, intra-individual comparison between brain tumor and contralateral brain tissue is widely adopted. So, the contralateral brain tissue can be easily segmented if both segmentations of brain and tumor have been achieved.

The models were optimized from the basic 3D U-Net architecture (Fig. 1E) [3]. The network weights were initially set with the Adam optimizer and a learning rate of 10^−4^. A loss function based on dice loss and focal loss was adopted to solve the issue of imbalanced class due to minor volume of ROI. The loss function put weights of 0.75 and 0.25 respectively on ROI and non-ROI voxels.

### Model training and validation

To train our 3D U-Net models [3], we first established a training dataset by random selection, with the remaining data as test dataset. Before the training, data preprocessing was performed, including intensity normalization and isotropic resampling by B-spline interpolation to isotropic 0.5 mm. Data were augmented by rotations for certain times of 90 degrees, vertical and horizontal flips. Since previous studies have illustrated that a bigger patch size is generally associated with superior model performance [6, 9], here, a patch size of 64 × 64 × 64 is selected due to the trade-off between run time, resource constraint and information loss. To confirm the applicability in different MRI settings, training and validation were performed in two MRI datasets: Leuven and TCIA datasets, with noise-added images for extra validation (Fig. 1D) [4, 12]. For the noise addition, Gaussian white noise was added with different levels of sigma values from 1 to 15, with a step of 1 after normalization of images into the range 0–255. Contralateral normal brain tissue and background areas on T2WI images were selected for the calculation of signal–noise-ratio (SNR) (Additional file 1: Fig. S1). The SNR for both datasets was decreased to 1, when the sigma value is around 15 (Additional file 1: Fig. S2).

### Quantitative evaluation of AI performance

Dice similarity coefficient (DSC) was adopted to quantify the volume-based similarity between ground truth and AI-derived segmentation [29]. The overlapping area between them is proportional to the DSC value, which is always between 0 and 1. Volume ratio (RV) computes the ratio of the ROI volumes from two segmentations, defined as RV (seg1, seg2) = V1/V2, where V1 and V2 are the volumes of two segmentations. Mean surface distance (MSD) and Hausdorff distance (HD) are designed to measure the surface-based difference between two segmentations [7]. MSD computes the average distance between the two segmentation surfaces, whereas HD computes the largest distance between them.

$$ \begin{aligned} & Dice\,Similarity\,Coefficient = \frac{{2\left| {P \cap G} \right|}}{\left| P \right| + \left| G \right|} \\ & Volume\,ratio = \frac{{V_{1} }}{{V_{2} }} \\ & Mean\,surface\,distance = \frac{1}{{n_{S} + n_{{S^{\prime}}} }}\left( {\mathop \sum \limits_{p = 1}^{{n_{S} }} d\left( {p,S^{\prime}} \right) + \mathop \sum \limits_{{p^{\prime} = 1}}^{{n_{{S^{\prime}}} }} d(p^{\prime},S)} \right) \\ \end{aligned} $$

where p: pixel; S, S′: surface of model segmentation and ground truth, d(p, S′): minimum Euclidean distance between p and all pixels p′ on surface S′

$$ Hausdorff\,distance = max\left\{ {\begin{array}{*{20}c} {sup} \\ {x \in X} \\ \end{array} d\left( {x, Y} \right),\begin{array}{*{20}c} { sup} \\ {y \in Y} \\ \end{array} d\left( {X,y} \right)} \right\} $$

### Practicability of AI-assisted segmentation

To illustrate whether AI-assisted segmentation can reduce the inter-observer disparity, inter-observer disparity on fully human segmentations was compared with the disparity of AI-assisted segmentations. The inter-observer disparity was calculated by comparing the difference of native masks from two radiologists (Yicheng Ni and Yuanbo Feng). While the disparity of AI-assisted segmentations was calculated by comparing masks that were first generated by AI models and then modified by the two radiologists. Additionally, the time between de novo manual segmentation and AI-assisted segmentation was also compared.

## Results

### Exemplified images from Leuven and TCIA datasets

**Fig. 2:**
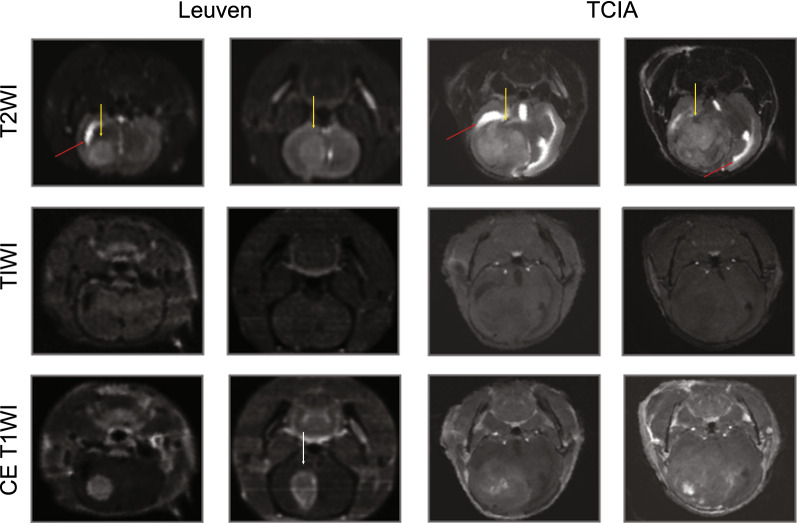
Exemplified images of Leuven and TCIA database. Images of T2WI, T1WI and CE-T1WI from both Leuven dataset (left) and TCIA dataset (right). Diversity in these radiological characteristics like ventriculomegaly, tumor, and intratumoral perfusion deficiency are indicated by red, yellow and white arrows respectively

As shown in Fig. 2, hyperintense brain tumor was observed on T2WI and CE-T1WI, with hypointense lesions on T1WI, compared with contralateral brain tissue in both Leuven and TCIA datasets. Additionally, tumor occupying signs like ventriculomegaly, and midline-shift of the brain were also observed. However, there were some disparities between the two datasets, in terms of scanning parameters, signal intensity after contrast agent injection, field of view, and animal species (Table 1). Specifically, in Leuven dataset, the entire head and neck region was captured with larger head sizes and more clearly bordered tumors, compared with TCIA dataset. Most cases in TCIA dataset are poorly enhanced on CE-T1WI, compared with all well-enhanced CE-T1WI in Leuven dataset. The volume ratio between tumor and brain is higher in cases from the TCIA dataset, compared with cases from the Leuven dataset. Leuven dataset has a better signal noise ratio (SNR) than TCIA (Additional file 1: Fig S2).

### Models training and validation

**Fig. 3:**
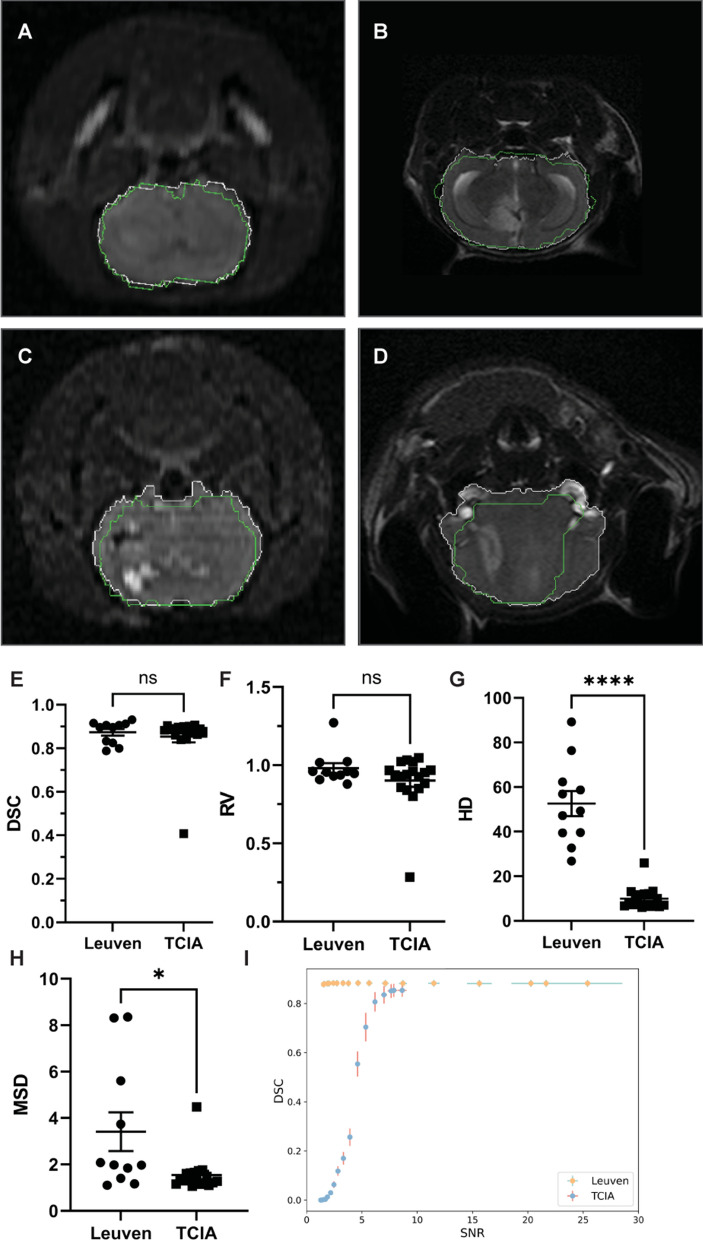
Segmentation of tumor-bearing brain in both datasets. The best and the worst prediction on Leuven dataset (**A**, **C**) and TCIA dataset (**B**, **D**) were shown. Ground truth and AI predicted segmentation were shown in T2WI MRI images in white and green respectively. Comparison of AI model performance between Leuven and TCIA datasets were finished by paired t tests on DSC, RV, HD and MSD (**E**–**H**). Performance of AI model after addition of different levels of Gaussian noise between Leuven and TCIA dataset were also shown (**I**). Data here are showed as mean ± standard error of mean. Abbreviations: DSC: Dice similarity coefficient; RV: relative ratio; HD: Hausdorff distance; MSD: mean surface distance; SNR: signal–noise ratio, ns: non-significant, *: < 0.05, ****: < 0.0001

Two models were trained and validated in a sequential order with similar methodology (Fig. 1C). Model 1 reached convergence during the first 5 epochs compared with model 2 which reached convergence around 15 epochs (Additional file 1: Fig S3). The best and the worst performance of model 1 were shown (Fig. 3A–D). The performance of model 1 in both datasets was comparable as measured by DSC (0.873 vs. 0.854, *p* > 0.05) and RV (0.981 vs. 0.902, *p* > 0.05). The worst performance was observed in a case with a large tumor and significant tumor occupying sign (Fig. 3D). Additionally, we also observed a higher HD (52.590 vs. 9.957, *p* < 0.0001) and MSD (3.415 vs. 1.543, *p* < 0.05) values in Leuven dataset than in TCIA (Fig. 3G, H). In Gaussian noise challenge, segmentation performance of model 1 remained unchanged in the Leuven dataset, when the SNR was greater than two (Fig. 3I). However, the performance of model 1 in TCIA dataset could be unaffected only if the SNR was higher than eight. The model performances on two datasets with noise challenge were further confirmed by RV, MSD and HD (Additional file 1: Fig S4A–C).

**Fig. 4:**
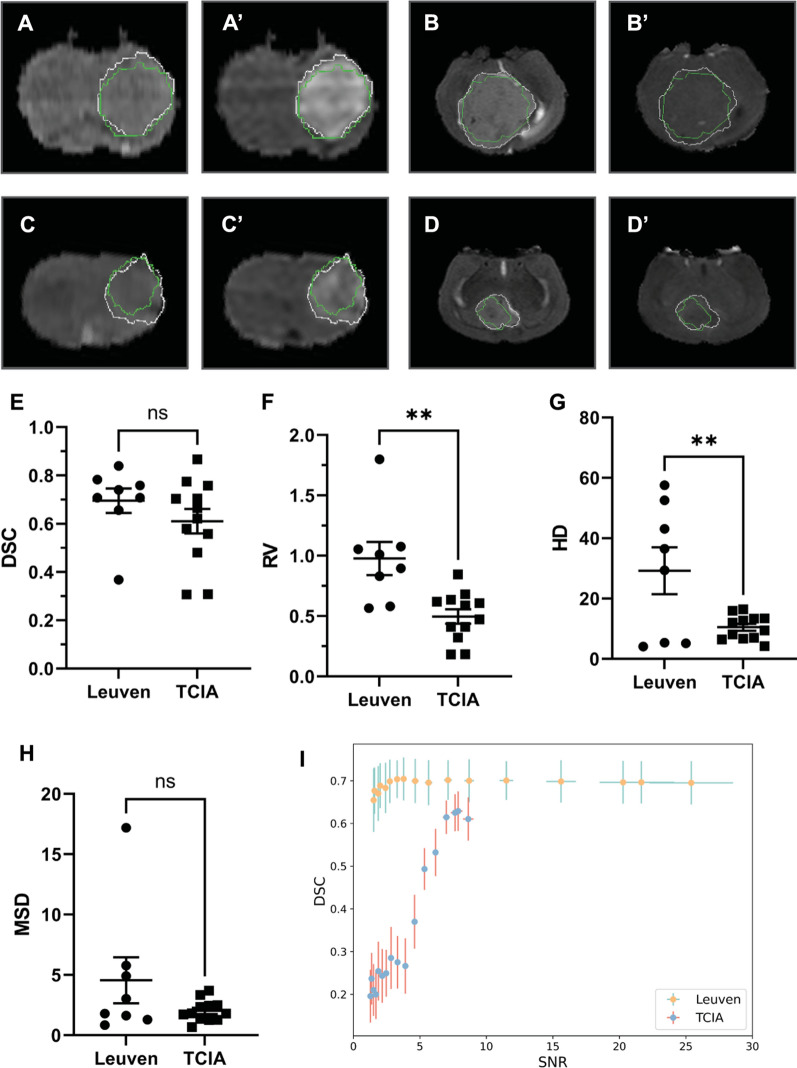
Segmentation of tumor in both datasets. The best prediction on Leuven dataset by T2WI (**A**) and CE-T1WI (**A**′) and TCIA dataset by T2WI (**B**) and CE-T1WI (**B**′). The worst prediction on Leuven dataset by T2WI (**C**) and CE-T1WI (**C**′) and TCIA dataset by T2WI (**D**) and CE-T1WI (**D**′). Ground truth and AI predicted segmentation are plotted in white and green respectively. Comparison of AI model performance between Leuven and TCIA datasets were finished by paired t tests on DSC, RV, HD and MSD (**E**–**H**). Performance of AI model after addition of different levels of Gaussian noise between Leuven and TCIA dataset (**I**). Data here are showed as mean ± standard error of mean. Abbreviations: DSC: Dice similarity coefficient; RV: relative ratio; HD: Hausdorff distance; MSD: mean surface distance; SNR: signal–noise ratio; ns: non-significant, **: < 0.01

Segmentation of brain tumor from brain images by model 2 was successfully achieved in each applied dataset, with marginally inferior performance in TCIA dataset as measured by DSC (0.610 vs. 0.695, *p* > 0.05) and RV (0.497 vs. 0.977, *p* < 0.01) (Fig. 4A–F). Similarly, significantly higher HD (29.222 vs. 10.485, *p* < 0.01) was observed in Leuven dataset, however, MSD (4.554 vs. 2.017, *p* > 0.05) did not differ significantly between two datasets. The inferiority was usually observed in cases with poorly perfused tumor lesions, larger tumor size and significant tumor accompanying signs like ventriculomegaly. After adding different levels of noise, the performance of model 2 remained uncompromised even when the SNR was close to three in Leuven dataset. However, model 2 only segmented well when the SNR is higher than eight in TCIA dataset (Fig. 4I, Additional file 1: Fig S4D–F).

### AI-assisted segmentation

**Fig. 5:**
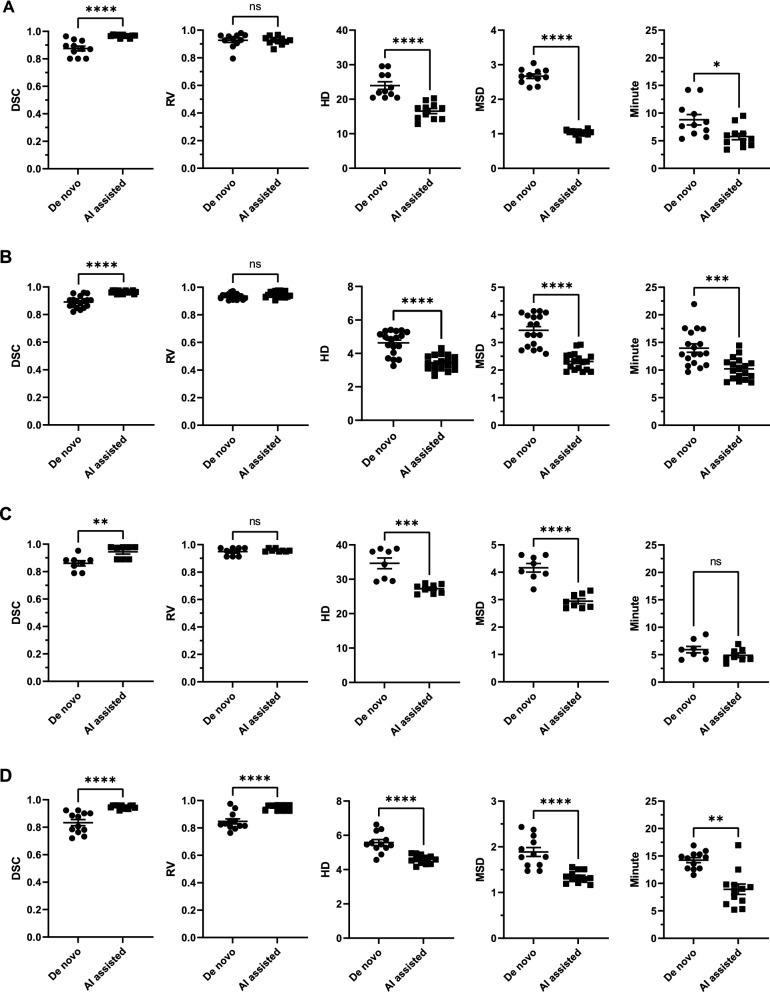
Quantitative evaluation on AI-assisted segmentation. DSC, RV, HD and MSD of inter-observer disparity based on fully manual segmentation and inter-observer disparity based on AI assisted segmentation in Leuven dataset (**A**) and TCIA dataset (**B**) for model 1, with model 2 in Leuven dataset (**C**) and TCIA dataset (**D**). Data here are showed as mean ± standard error of mean. Abbreviations: DSC: Dice similarity coefficient; RV: relative ratio; HD: Hausdorff distance; MSD: mean surface distance; ns: non-significant; *: < 0.05; **: < 0.01; ***: < 0.001; ****: < 0.0001

For model 1, AI-assisted segmentation yielded less diverse results as measured by DSC (0.875 vs. 0.966, *p* < 0.0001), HD (23.949 vs. 16.559, *p* < 0.0001), MSD (2.668 vs. 1.031, *p* < 0.0001), and reduced the segmentation time (8.812 vs. 5.750, *p* < 0.05) for Leuven dataset (Fig. 5A). Similarly, these could also be observed in application of the model 1 in TCIA dataset, as indicated by DSC (0.891 vs. 0.964, *p* < 0.001), HD (4.626 vs. 3.442, *p* < 0.0001), MSD (3.444 vs. 2.313, *p* < 0.0001) and segmentation time (13.974 vs. 10.221, *p* < 0.001) (Fig. 5B). Similarly, AI-assisted segmentation pipeline of model 2 helped reduce the inter-observer disparity in Leuven dataset, compared with fully manual segmentation, as indicated by DSC (0.861 vs. 0.944, *p* < 0.0001), HD (34.637 vs. 27.245, *p* < 0.0001), MSD (4.164 vs. 2.945, *p* < 0.0001) (Fig. 5C). Segmentation time has reduced significantly (5.934 vs. 4.887, *p* < 0.05). The improvement in segmentation consistence by model 2 was also observed in TCIA dataset, as indicated by DSC (0.833 vs. 0.947, *p* < 0.0001), HD (5.576 vs. 4.599, *p* < 0.0001), MSD (1.886 vs. 1.342, *p* < 0.0001) (Fig. 5D). AI-assisted segmentation is associated with shorter segmentation time (8.931vs.14.239, *p* < 0.001).

In model 1, most of correction mainly involved brain-skull border, misclassification of cranial nerves, and/or labyrinth. Extremely poor performance was mainly observed in TCIA cases with larger tumor volume, greatly changed brain anatomy, and spread of tumor into skull. In model 2, tumor border was the most modified area.

## Discussion

This study has met its preset aims: training and validation of 3D U-Net based models for automatic segmentation of tumor bearing brains and brain tumor lesions, based on datasets from two research centres. Furthermore, the performance of these models has been validated in images with low SNR, which ensures its application in low quality image data. These models may assist quantitative imaging analyses, surgical planning and 3D printing by reducing inter-observer disparity and segmentation time.

The generalizability and representability of models here are based on the following facts. Firstly, this study adopted different tumor models, with implanted tumor in rats in Leuven dataset and primary brain tumor in genetically modified mice and implanted tumor model in mice in TCIA dataset. Secondly, MRI data were acquired with commonly used sequences including T2WI, T1WI and CE-T1WI at 3.0T magnets. Thirdly, different scanning settings among multi-center data, including scanning parameters and field of view, represent the practical scenarios of future applications. Lastly, models’ robustness was tested with Gaussian noise addition.

U-Net is a neural network dedicated for ROI delineation. The 3D U-Net architecture, an updated version of 2D U-Net, can interpret the cross-plane spatial information based on the same encoder-decoder structure of its 2D counterpart. Technically, 3D convolution followed by 3D max-pooling was adopted in its encoder path, with 3D up-sampling together with the spatial information during encoding in decoder path. This architecture has been tested in various scenarios of medical imaging, with robust performance [16, 21]. Additionally, it is noteworthy that novel variants of U-Net have been proposed, which are believed to be methodologically superior, including UNeXt, nnU-Net, cascaded U-Net, U-NetCC, double U-Net, and recurrent residual U-Net [1, 11, 13–15, 28]. The architecture may possibly improve the performance reported here, however, these newer variants have not been fully tested in application level and the retraining with these models is not the aim of current study.

Recently, quantitative imaging analyses in preclinical animal models have become increasingly important [8]. Radiomics can automatically extract image features like volume, shape, texture and signal intensity distribution, which mostly can reflect underlying tissue heterogeneity and pathophysiology. Proper segmentation is crucial for these features’ extraction, and slight changes in ROI introduced by inter-observer disparities may lead to significant changes in radiomics features and subsequent radiomics-based prediction [27]. Computer-aided segmentation may reduce the inter-observer disparity and thus produce more robust and reproducible radiomic features [8]. The models here, together with radiomics models developed in the future, will form an automated pipeline for molecular classification, prognostic prediction, and so on in preclinical animal study, ultimately facilitating clinical development.

Both model 1 and model 2 performed well in Leuven dataset, even after extensive Gaussian noise addition. The relatively poor prediction of model 1 in TCIA dataset can be accountable to the following facts. Firstly, after isotropic resampling the imaging volume for mice is lower than that in Leuven dataset. Secondly, the image quality is poorer than that of Leuven dataset, as indicated by initial SNR. Thirdly, anatomical distortion was found greater in TCIA cases due to large tumor size which disrupted the image texture. Lastly, most scans did not cover entire head region, and cerebellum was not included in the scanning region.

The model 2 generally yielded a poorer performance than model 1, as indicated by DSC, which can be partially attributed to the ambiguous tumor border. The ambiguous border even raised the disparity between human radiologists, as indicated by the inter-observer disparity in radiologists’ segmentations (Fig. 5). The poorer performance of model 2 in TCIA dataset, compared with Leuven dataset, can be explained additionally by the heterogenous and poor enhancement behavior in CE-T1WI and diffuse tumor borders.

With training model 1, due to the demanding hardware resources of 3D U-Net, the input shape was set as 64 × 64 × 64 × 3. After isotropic resampling and central cropping, the matrix size for Leuven dataset is 64 × 128 × 128 × 3, compared with 64 × 64 × 64 × 3 in the TCIA dataset. Thus, Leuven data were patchified into 64 × 64 × 64 × 3 before filling into the model, with TCIA data being natively filled. This explains the higher maximal false positive rate in validation of model 1 with Leuven data than with TCIA data (0.28 vs. 0.14), because cross cube interpretation of image texture is disabled during patchifying. The “counter-intuitive” significantly higher HD and MSD value in validation of Leuven dataset can be explained by its bigger matrix size. Thus, cautious interpretation of these parameters is suggested when comparing model performance in data with different matrix size. HD and MSD would be good measures when comparing ROIs based on the same dataset as we did in Fig. 5.

Despite encouraging segmentation performance here, the following limitations should be addressed. Firstly, the most important limitation is a lack of gold standard for manual segmentation, especially for tumor lesions in TCIA dataset. Secondly, AI-based auto-segmentation is a data-driven toolbox, thus, its performance on external real life use depends on the variety of data filled during training process. Trans-species use may not yield expected performance. Thirdly, these models were trained based on T1WI, CE-T1WI and T2WI data, thus, only cases with complete scan of these sequences are eligible for satisfactory automatic segmentation. Lastly, during AI-assisted segmentation, increased time for manual correction may be foreseeable for cases with extremely distorted anatomy in brain.

## Conclusion

We proposed 3D U-Net based models for auto segmentation of tumor-bearing brain and brain tumor lesion respectively, based on volumetric MRI data from rats and mice. The automated platforms demonstrated satisfactory delineation for brain and tumor respectively based on T1WI, CE-T1WI and T2WI images. Models here were further challenged with Gaussian noise addition and showed robust reproducibility in different settings as measured by quantitative measures. The application of AI-assisted segmentation can reduce interobserver disparity and thus present a possibility of automatic imaging analyses pipeline for translational animal studies. Hopefully, these tools may be of help for peers in quantitative imaging analyses, animal surgery planning, and 3D printing.

## Supplementary Information

**Additional file 1: Figure S1**. Segmentation of area for calculation of signal to noise ratio. Brain tissue without tumor and background area were selected in either orange or green in both Leuven (A) and TCIA (B) datasets. **Figure S2** Signal noise ratio changes after noise addition. Change of signal noise ratio by adding different levels of Gaussian noise evaluated by sigma value for both Leuven and TCIA datasets. Data here are showed as mean ± standard error of mean. Abbreviation: SNR: signal to noise ratio. **Figure S3** Model training process by loss value and intersection-over-union value. Loss values and intersection-over-union value for both training and validation processes in model 1 (A) and model 2 (B) by epoch were shown. **Figure S4** Quantitative evaluation of segmentation on noised images. Segmentation performance as measured by RV, HD and MSD under different SNR were shown for model 1 (A, B, C) and model 2 (D, E, F). Abbreviations: RV: relative ratio; HD: Hausdorff distance; MSD: mean surface distance; SNR: signal to noise ratio.

## Data Availability

Data from Mouse-Astrocytoma are available through The Cancer Imaging Archive at https://doi.org/10.7937/K9TCIA.2017.SGW7CAQW.
