# Supplementary material for: AI-based MRI auto-segmentation of brain tumor in rodents, a multicenter study
Source: Acta Neuropathol Commun. 2023 Jan 14;11:11. doi: 10.1186/s40478-023-01509-w (PMC9840251; doi:10.1186/s40478-023-01509-w)

**AI-based MRI auto-segmentation of brain tumor in rodents, a multicenter study**

Shuncong Wang^1#^, Xin Pang^1,5#^, Fredrick de Keyzer^2^, Yuanbo Feng^1^, Johan V. Swinnen^1^, Jie Yu^1^, Yicheng Ni^1^

^1^KU Leuven, Biomedical Group, Campus Gasthuisberg, Leuven 3000, Belgium; [shuncong.wang@kuleuven.be](mailto:shuncong.wang@kuleuven.be) (S. W.); pang.xin@hotmail.com (X. P.); [yicheng.ni@kuleuven.be](mailto:yicheng.ni@kuleuven.be) (Y. N.); [yuanbo.feng@kuleuven.be](mailto:yuanbo.feng@kuleuven.be) (Y. F.); [j.swinnen@kuleuven.be](mailto:j.swinnen@kuleuven.be) (J. S.); [j.yu@kuleuven.be](mailto:j.yu@kuleuven.be) (J. Y.).

^2^Department of Radiology, University Hospitals Leuven, KU Leuven, Herestraat 49, 3000 Leuven, Belgium; [frederik.dekeyzer@uzleuven.be](mailto:frederik.dekeyzer@uzleuven.be) (F. K.);

^3^Nuclear Medicine and Molecular Imaging, KU Leuven, Leuven, Belgium. [xikai.tang@kuleuven.be](mailto:xikai.tang@kuleuven.be) (X. T.).

^4^Medical Imaging Research Center (MIRC), KU Leuven, Leuven, Belgium.

^5^Current address: Diepenbrockstraat 21, 1077 VX Amsterdam, Netherlands.

^*^Correspondence: Yicheng Ni, MD, PhD, KU Leuven, Biomedical Group, Campus Gasthuisberg, Leuven 3000, Belgium; yicheng.ni@kuleuven.be (Y. N.).

^#^These authors contributed equally to the current study.

**Running title**: AI segmentation of brain tumor in rodents

**Supplementary figure 1. Segmentation of area for calculation of signal to noise ratio.** Brain tissue without tumor and background area were selected in either orange or green in both Leuven (A) and TCIA (B) datasets.

**
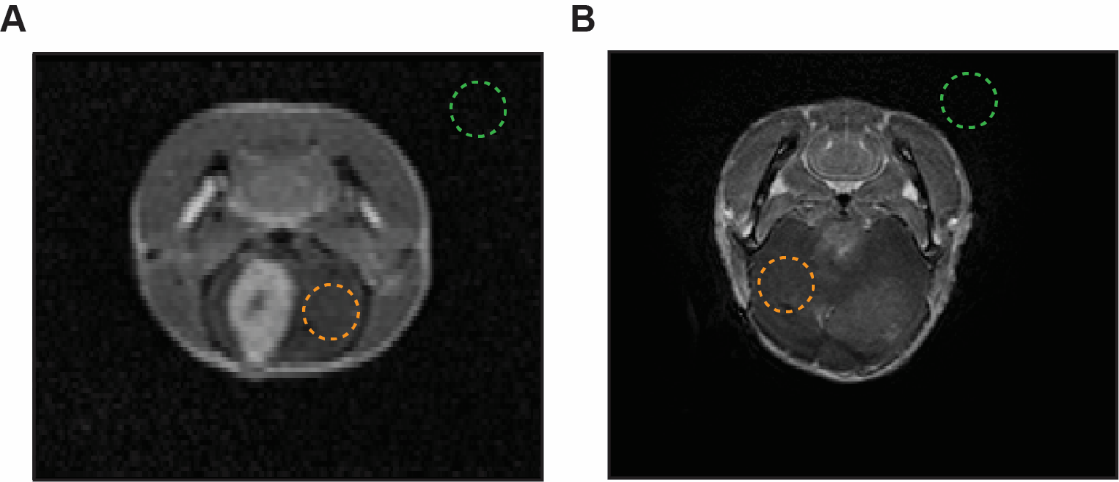
**

**Supplementary figure 2. Signal noise ratio changes after noise addition.** Change of signal noise ratio by adding different levels of Gaussian noise evaluated by sigma value for both Leuven and TCIA datasets. Data here are showed as mean ± standard error of mean. Abbreviation: SNR: signal to noise ratio.

**
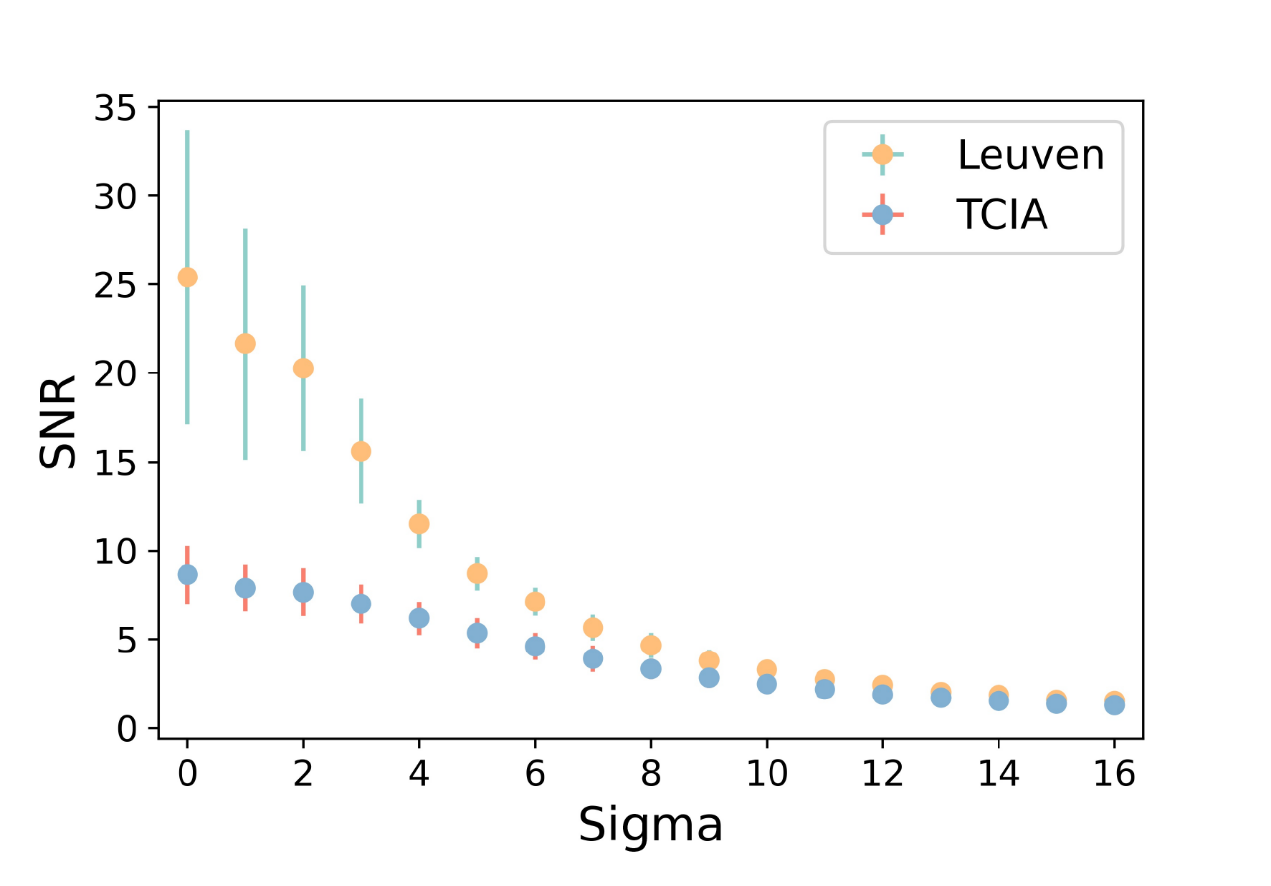
**

**Supplementary figure 3. Model training process by loss value and intersection-over-union value.** Loss values and intersection-over-union value for both training and validation processes in model 1 (A) and model 2 (B) by epoch were shown.
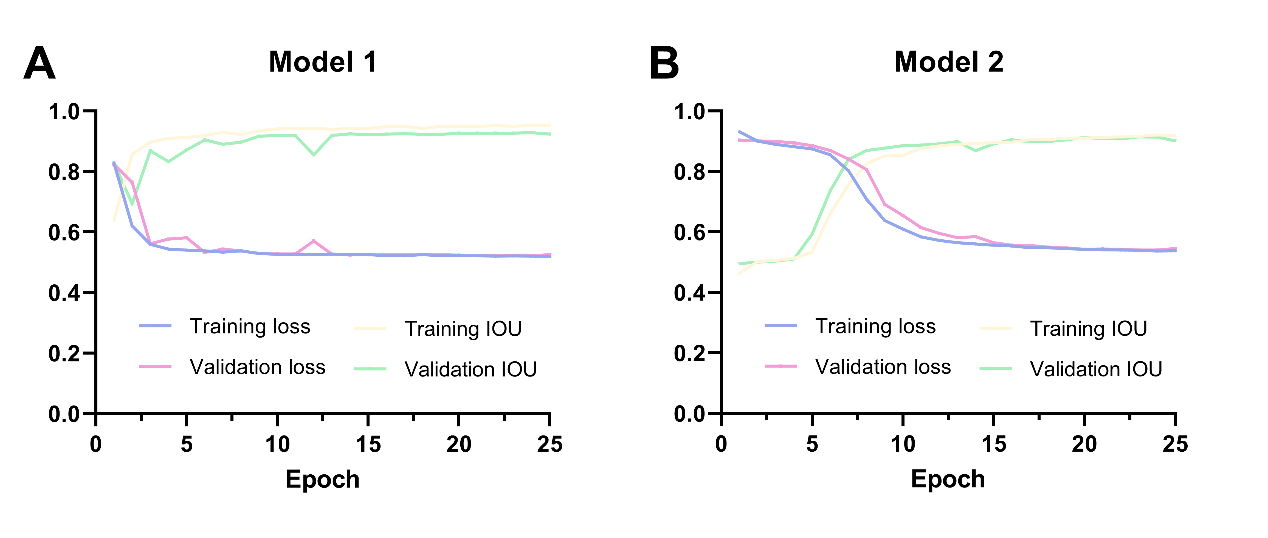


**Supplementary figure 4. Quantitative evaluation of segmentation on noised images.** Segmentation performance as measured by RV, HD and MSD under different SNR were shown for model 1 (A, B, C) and model 2 (D, E, F). Abbreviations: RV: relative ratio; HD: Hausdorff distance; MSD: mean surface distance; SNR: signal to noise ratio.
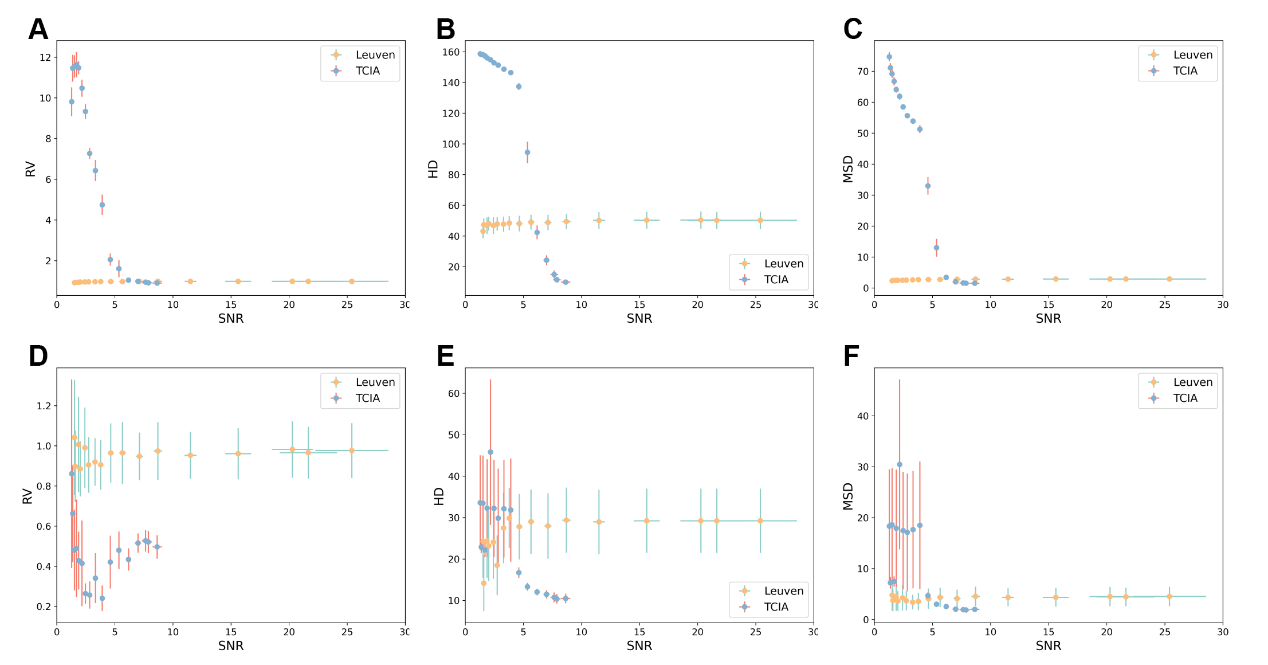

Supplement: Supplementary file 1 — Additional file 1: Figure S1. Segmentation of area for calculation of signal to noise ratio. Brain tissue without tumor and background area were selected in either orange or green in both Leuven (A) and TCIA (B) datasets. Figure S2 Signal noise ratio changes after noise addition. Change of signal noise ratio by adding different levels of Gaussian noise evaluated by sigma value for both Leuven and TCIA datasets. Data here are showed as mean ± standard error of mean. Abbreviation: SNR: signal to noise ratio. Figure S3 Model training process by loss value and intersection-over-union value. Loss values and intersection-over-union value for both training and validation processes in model 1 (A) and model 2 (B) by epoch were shown. Figure S4 Quantitative evaluation of segmentation on noised images. Segmentation performance as measured by RV, HD and MSD under different SNR were shown for model 1 (A, B, C) and model 2 (D, E, F). Abbreviations: RV: relative ratio; HD: Hausdorff distance; MSD: mean surface distance; SNR: signal to noise ratio. [file 40478_2023_1509_MOESM1_ESM.docx]
